# Effects of postnatal interventions for the reduction of vertical HIV transmission on infant growth and non-HIV infections: a systematic review

**DOI:** 10.7448/IAS.16.1.18865

**Published:** 2013-12-20

**Authors:** Moleen Zunza, Gareth D Mercer, Lehana Thabane, Monika Esser, Mark F Cotton

**Affiliations:** 1 Department of Paediatrics and Child Health, Children's Infectious Disease Clinical Research Unit, Stellenbosch University, Tygerberg, South Africa; 2 MD/PhD Program, Faculty of Medicine, University of British Columbia, Vancouver, British Columbia, Canada; 3 Department of Clinical Epidemiology and Biostatistics, McMaster University, Hamilton, Ontario, Canada; 4 Biostatistics Unit/FSORC, St Joseph's Healthcare Hamilton, Hamilton, Ontario, Canada; 5 Department of Pathology, Immunology Unit, NHLS, Stellenbosch University, Tygerberg, South Africa

**Keywords:** postnatal interventions, HIV, children, growth, non-HIV infections, breast milk

## Abstract

**Introduction:**

Guidelines in resource-poor settings have progressively included interventions to reduce postnatal HIV transmission through breast milk. In addition to HIV-free survival, infant growth and non-HIV infections should be considered. Determining the effect of these interventions on infant growth and non-HIV infections will inform healthcare decisions about feeding HIV-exposed infants. We synthesize findings from studies comparing breast to formula feeding, early weaning to standard-duration breastfeeding, breastfeeding with extended antiretroviral (ARV) to short-course ARV prophylaxis, and alternative preparations of infant formula to standard formula in HIV-exposed infants, focusing on infant growth and non-HIV infectious morbidity outcomes. The review objectives were to collate and appraise evidence of interventions to reduce postnatal vertical HIV transmission, and to estimate their effect on growth and non-HIV infections from birth to two years of age among HIV-exposed infants.

**Methods:**

We searched PubMed, SCOPUS, and Cochrane CENTRAL Controlled Trials Register. We included randomized trials and prospective cohort studies. Two authors independently extracted data and evaluated risk of bias. Rate ratios and mean differences were used as effect measures for dichotomous and continuous outcomes, respectively. Where pooling was possible, we used fixed-effects meta-analysis to pool results across studies. Quality of evidence was assessed using the GRADE approach.

**Results and discussion:**

Prospective cohort studies comparing breast- versus formula-fed HIV-exposed infants found breastfeeding to be protective against diarrhoea in early life [risk ratio (RR)=0.31; 95% confidence interval (CI)=0.13 to 0.74]. The effect of breastfeeding against diarrhoea [hazard ratio (HR)=0.74; 95% CI=0.57 to 0.97] and respiratory infections (HR=0.65; 95% CI=0.41 to 1.00) was significant through two years of age. The only randomized controlled trial (RCT) available showed that breastfeeding tended to be protective against malnutrition (RR=0.63; 95% CI=0.36 to 1.12). We found no statistically significant differences in the rates of non-HIV infections or malnutrition between breast-fed infants in the extended and short-course ARV prophylaxis groups.

**Conclusions:**

Low to moderate quality evidence suggests breastfeeding may improve growth and non-HIV infection outcomes of HIV-exposed infants. Extended ARV prophylaxis does not appear to increase the risk for HIV-exposed infants for adverse growth or non-HIV infections compared to short-course ARV prophylaxis.

## Introduction

HIV infection among children is a public health concern especially in poorly resourced countries [1]. Most children acquire HIV infection through mother-to-child transmission (MTCT) [2]. Approximately 2.5 million children are living with HIV/AIDS worldwide [3]. Although the proportion of HIV-attributable death among children less than five years of age is declining worldwide, HIV/AIDS is still a leading cause of premature death in Southern African children [4]. In the absence of antiretroviral (ARV) treatment, one third of HIV-infected children die by one year of age and about 50% by two years of age [4]. Infectious diseases and nutritional complications are the predominant underlying causes of mortality in these children [4].

HIV may be vertically transmitted in pregnancy, labour, delivery, or through breast milk. Without interventions, 15–30% of infants are vertically infected; breastfeeding increases the risk to 20–45% [5]. Strategies to reduce postnatal vertical transmission of HIV focus on reducing transmission through breast milk. HIV-positive mothers in high-income countries are recommended to completely avoid breastfeeding [6]. However, in poorly resourced countries where formula feeding does not generally meet AFASS criteria (Acceptable, Feasible, Affordable, Sustainable, and Safe), avoiding breastfeeding increases the risks of infant mortality and infectious morbidity [7].

### Description of the intervention

The efficacy of ARV regimens in reducing HIV vertical transmission through breast milk has been demonstrated in several randomized controlled trials (RCTs) [8–11], these interventions have since been incorporated into the World Health Organization (WHO) guidelines on infant feeding by HIV-positive mothers [12].

WHO 2013 prevention of MTCT (PMTCT) guidelines recommend that all HIV-positive pregnant women receive highly active ARV treatment (HAART), until at least one week after cessation of breastfeeding or after delivery when formula feeding, but should preferably be continued as lifelong therapy regardless of CD4 count [12]. Mothers with CD4 count ≤500cells/mm^3^ or WHO clinical stage 3 or 4 disease are recommended to continue lifelong ARVs. HIV-exposed infants on breast milk are recommended to receive once-daily nevirapine (NVP) prophylaxis until they are fully weaned. Formula-fed infants should receive 4–6 weeks of daily NVP or twice-daily zidovudine (ZDV) [12].

### Effect of postnatal MTCT interventions on infant growth and non-HIV infections

Compared to infant formula, breast milk protects against gastrointestinal and respiratory tract infections and improves overall survival [13]. Breastfeeding also promotes optimal child growth until two years of age [14].

ARVs drugs minimize postnatal HIV transmission through breast milk by reducing breast milk viral load. As ARVs have clinical and laboratory adverse effects, their safety in HIV-exposed children should be considered. Baroncelli *et al*. reported a high risk of anaemia in HIV-exposed infants exposed to HAART with ZDV alone compared to HAART without ZDV, which disappeared at one month of life [15]. Grade 3–4 hepatoxicity was reported in infants exposed to NVP for at least five days [16]. Neonatal exposure to lopinavir/ritonavir (LPV/r) has been associated with cardiac toxicity and adrenal dysfunction [17]. Lamivudine exposure is safe in HIV-exposed infants [18]. While side effects would not negate the benefits of ARVs in preventing HIV transmission, it is important for health policy makers to have accurate estimates of the anticipated risks of such effects when introducing these interventions into clinical practice.

### Why it is important to do this review

A Cochrane review appraised evidence for the efficacy of postnatal HIV PMTCT interventions in preventing HIV transmission, and improving HIV-free survival [19]. However, in addition to their efficacy in preventing HIV transmission, policy makers should consider the effects of these interventions on infant growth and susceptibility to non-HIV infections.

Contradictory findings of the effects of different postnatal PMTCT interventions on infant growth and non-HIV infectious morbidity were reported in clinical trials and observational studies; therefore the true effects of the interventions on these outcomes are uncertain. To inform decision-making about HIV PMTCT recommendations, this review aims to synthesize findings from studies comparing the effects of different postnatal interventions for PMTCT of HIV on infant growth and non-HIV infections, with follow-up periods of between 3 and 24 months of age.

### Objectives

To collate and appraise evidence of interventions to reduce postnatal vertical HIV transmission in HIV-exposed infants, and estimate their effect on (1) growth from birth to two years of age (primary objective) and (2) non-HIV infections from birth to two years of age (secondary objective).

## Methods

### Criteria for considering studies for this review

#### Studies

- RCTs of postnatal interventions to prevent vertical transmission of HIV, which included the assessment of infant growth or non-HIV infections.
- RCTs assessing the effect of established postnatal interventions for prevention of vertical transmission of HIV on infant growth or on HIV infections.
- Cohort studies were also included if the intervention (e.g. mode of feeding) could not be ethically randomized.

#### Participants

HIV-positive mothers and their infants.

#### Interventions

Intervention aimed at reducing HIV vertical transmission.

#### Primary outcomes

- Weight-for-age (WAZ), weight-for-length (WLZ), length-for-age (LAZ), and head circumference-for-age (HCA) *z-*scores and malnutrition.
- Non-HIV infections, e.g. respiratory tract infections, gastrointestinal infections.

### Search methods for identification of studies

#### Electronic searches

Search strategies developed by The Cochrane Collaboration HIV/AIDS Review group were used to search for studies [19]. PubMed (24 April 2013), SCOPUS (24 April 2013), and Cochrane CENTRAL Controlled Trials Register (11 March 2013) were searched without language, time or publication status restrictions (Supplementary file). Dates indicate the time when searches were last performed in each database. The reference lists of included studies were searched for studies.

### Data collection and analysis

#### Selection of studies

Two reviewers (MZ and GM) independently reviewed abstracts of electronic search results. Full texts of potentially relevant articles were retrieved and independently examined for eligibility.

#### Data extraction

The following data were independently extracted in duplicate: study design, study duration, methodological quality, study interventions, and outcomes. Discrepancies were resolved through discussion.

#### Assessment of risk of bias in included studies

The Cochrane Collaboration's risk of bias tool was used to assess the methodological quality of each selected study [20]. Two authors (MZ and GM) independently assessed the risk of bias. The following domains were assessed: sequence generation; allocation concealment; blinding of participants, personnel, and outcome assessors; whether incomplete outcomes data were adequately addressed; selective reporting; and other bias.

$$\begin{array}{c} \text{SD}=\sqrt{\frac{\left(N_{1}-1\right)SD_{1}^{2}+\left(N_{2}-1\right)SD_{2}^{2}+\frac{N_{1}N_{2}}{N_{1}+N_{2}}\left(M_{1}^{2}+M_{2}^{2}-2M_{1}M_{2}\right)}{N_{1}+N_{2}-1}}\\ \frac{Mean=N_{1}+M1+N_{2}M_{2}}{N_{1}+N_{2}} \end{array}$$

ClinicalTrial.gov and Current Controlled trial registries were searched for protocols of included studies. If the protocol was unavailable, the methods and results sections were compared to assess the potential for selective reporting bias.

#### Measures of treatment effect

When included publications presented summary data separately for each intervention group, we calculated risk ratios (RR) for binary outcomes and mean differences (MD) for continuous outcomes, and associated 95% confidence intervals (CI). Otherwise we have directly presented the effect estimates [RR, hazard ratio (HR), and odds ratios (OR)] reported in the publications.

For infectious morbidity events, we assumed that the occurrence of each outcome per participant is a random variable following a Poisson distribution. The normal approximation to the Poisson distribution was used to calculate CI for MD in the incidence of infectious morbidity outcomes. The 95% CI for MD was calculated as:

$\left({\hat{\lambda }}_{1}-{\hat{\lambda }}_{2}\right)\pm 1.96\times \sqrt{\frac{{\hat{\lambda }}_{1}}{n_{1}}+\frac{{\hat{\lambda }}_{2}}{n_{2}}}$ where ${\hat{\lambda }}_{1}$ and ${\hat{\lambda }}_{2}$ are estimated average counts of a specific infection in Groups 1 and 2, and *n*_1_ and *n*_2_ are the numbers of infants with complete follow-up data in each group [21]. Included studies did not report results disaggregated by sex separately for each study randomisation arm. Therefore, we could not extract study results disaggregated by sex in this review.

#### Unit of analysis issues

*Repeated observations on participants.* When results were presented for more than one time point, the following approaches were used to obtain single effect measures:

For infectious events, we computed the total number of events experienced during the entire follow-up period for each intervention group. For growth outcomes, summary data were extracted at the longest follow-up time point.

*Multiple intervention groups.* Experimental intervention groups deemed sufficiently comparable were combined for pairwise comparison with the control group. For dichotomous outcomes, sample size and outcome events were summed across combined groups. For continuous outcomes, means and standard deviations (SD) were combined using the following formulas [20]:

where *N*_1_, *M*_1_ and *SD*_1_ are sample size, mean and standard deviation of Group 1, *N*_2_, *M*_2_, *SD*_2_ are the corresponding values of Group 2.

*Studies using a factorial design.* One report was from a trial that used a factorial design [11]. We only report on the effect of ARV interventions in this review. Reports of the study did not suggest an important interaction between the two interventions.

#### Dealing with missing data

Authors of 12 studies were contacted for missing information; the requested information being obtained for six studies. The potential impact of missing data was considered during risk of bias assessment. Meta-analysis was repeated, excluding studies with attrition rates >20% to assess the robustness of the results to missing data, and both estimates are presented.

#### Assessment of heterogeneity

Substantial statistical heterogeneity was defined as an I^2^ statistic >50% [20].

#### Assessment of publication bias

Too few studies were included in each comparison to enable an investigation of publication bias.

#### Data synthesis

Fixed-effects meta-analysis using the Mantel-Haenszel method for dichotomous outcomes and the inverse-variance method for continuous outcomes were used to pool results across studies [20]. Where meta-analysis was inappropriate, individual study results were reported separately. Review Manager 5.1 was used for analysis.

### Quality of evidence

The Grades of Recommendation Assessment, Development and Evaluation (GRADE) approach was used to rate quality of evidence [22]. In evaluating the quality of RCT evidence, we considered the following in whether to downgrade the quality of evidence: methodological limitations, inconsistency in study results, indirectness, imprecision and publication bias. For observational studies, we considered the following factors in determining whether to upgrade the quality of evidence: large observed effect and whether plausible confounding would change the intervention effect. Our ratings for the breastfeeding versus formula feeding and the breastfeeding with extended versus short-course ARV prophylaxis comparisons are presented in Tables 1 and 2, respectively.

**Table 1:** Breastfeeding compared to formula feeding for HIV-exposed infants

Outcomes	Relative effect (95% CI)	Number of participants (studies)	Quality of the evidence (GRADE)
Malnutrition RCT	RR 0.63 (0.36 to 1.12)	371 (1)	⊕⊕⊖⊖ low^a^^,^ b
Diarrhoea “Cohort study, effect up to two years of age”	HR 0.74 (0.57 to 0.97)	557 (1)	⊕⊕⊕⊖ moderate^a^^,^ c
Diarrhoea “Cohort study, effect up to three months of age”	RR 0.31 (0.13 to 0.74)	127 (1)	⊕⊕⊖⊖ low^c^^,^ d
Respiratory infections RCT	HR 1 (0.9 to 1.11)	371 (1)	⊕⊕⊖⊖ low^a^^,^ b
Respiratory infections Cohort	HR 0.60 (0.36 to 0.98)	557 (1)	⊕⊕⊕⊖ moderate^c^
Diarrhoea RCT	HR 1.11 (0.91 to 1.43)	371 (1)	⊕⊕⊖⊖ low^a^^,^ b

a Study had some methodological limitations

b wide confidence interval and fails to exclude the null effect

c observed breastfeeding effect was considered clinically important

d sample size was too small. RR: Risk ratio; HR: Hazard ratio.

**Table 2:** Breastfeeding with extended ARV compared to breastfeeding with short-course ARV prophylaxis for HIV-exposed infants

	Illustrative comparative risks^a^ (95% CI)				
				
	Assumed risk	Corresponding risk			
Outcomes	Breastfeeding with short-course ARVs	Breastfeeding with extended ARVs	Relative effect (95% CI)	Number of participants (studies)	Quality of the evidence (GRADE)
	Study population				
Growth faltering	32 per 1000	36 per 1000 (27 to 48)	RR 1.12 (0.83 to 1.5)	5719 (3)	⊕⊕⊕⊖ moderate^b^^,^^c^
Pneumonia	The average incidence of pneumonia ranged across control groups from 0.03 to 0.11	The average incidence of pneumonia in the intervention groups was 0.01 lower (0.02 lower to 0.00 higher)		6437 (4)	⊕⊕⊕⊖ moderate^b^^,^^d^
Meningitis	The average incidence of meningitis ranged across control groups from 0.0089 to 0.0147	The average incidence of meningitis in the intervention groups was 0 higher (0.01 lower to 0.00 higher)		4914 (2)	⊕⊕⊖⊖ low^b^^,^^e^
Gastroenteritis	The average incidence of gastroenteritis ranged across control groups from 0.02 to 0.07	The average incidence of gastroenteritis in the intervention groups was 0.01 higher (0.01 lower to 0.02 higher)		6437 (4)	⊕⊕⊕⊖ moderate^b^^,^^c^

The assumed risk was based on the mean control group risk if there was one study included or otherwise, on mean range in control group risk across studies

a The corresponding risk (and its 95% CI) is based on the assumed risk in the comparison group and the relative effect of the intervention (and its 95% CI).

b There were too few studies to assess publication bias

c CI failures to exclude appreciable harm

d point estimates vary widely

e there were very few events. CI: Confidence interval; RR: Risk ratio.

## Results

### Included studies

**Figure 1:**
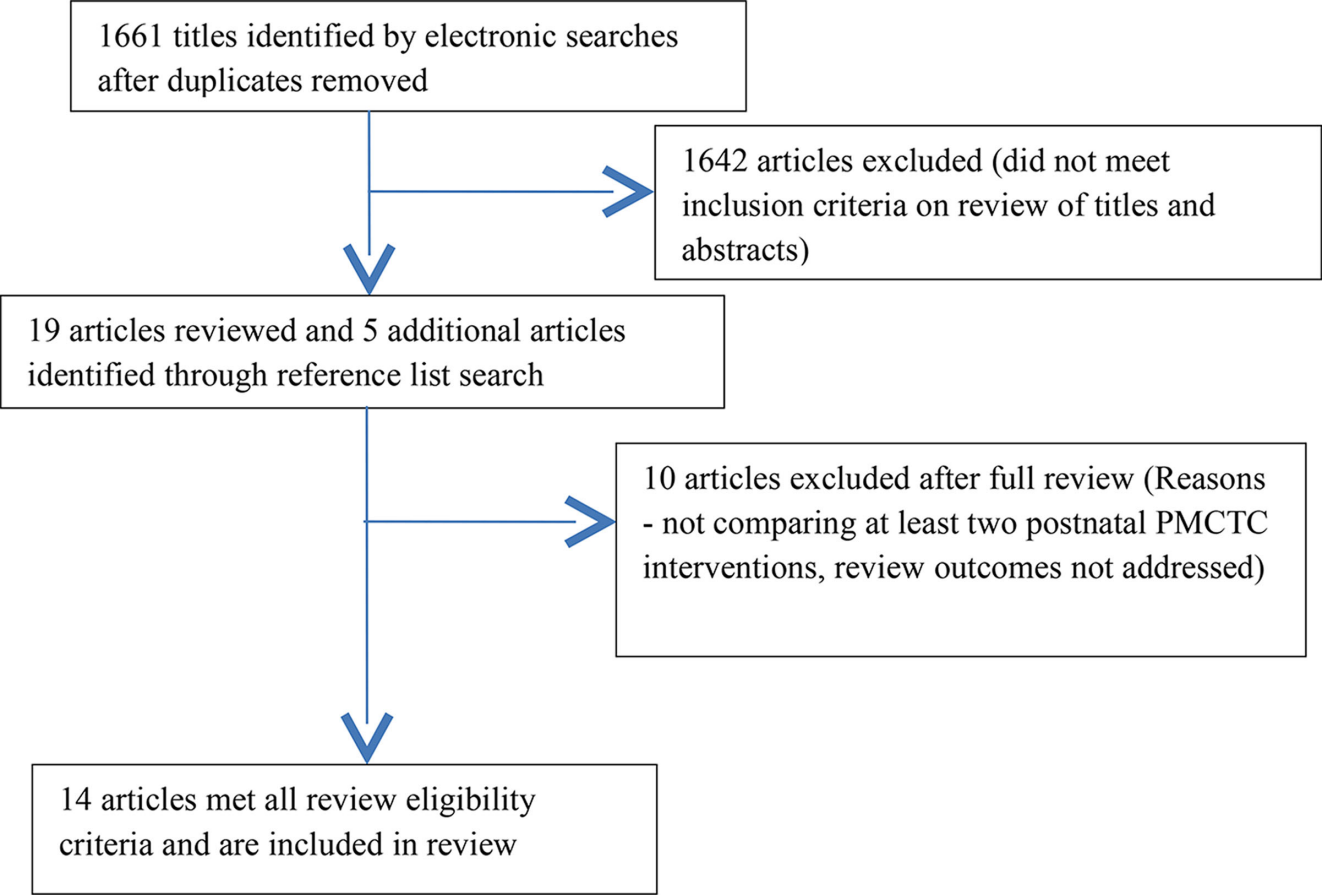
Flow diagram of screening process.

We identified 14 reports from seven RCTs and three prospective cohort studies (Figure 1) conducted in: South Africa (3), Zambia (1), Malawi (1), USA and Brazil (1), Cote d'lvoire (1), Tanzania (1), Kenya (1), Burkina Faso, Kenya and South Africa (1) and South Africa, Tanzania, Uganda and Zimbabwe (1) (Supplementary file). Ten studies were excluded on review of full articles (Supplementary file).

### Types of interventions

Table 3 summarizes the studies included and outcomes assessed under each comparison.

**Table 3:** Summary of included studies and outcomes assessed for each comparison

Comparisons	Studies (Sample size)	Outcomes assessed	Studies
Breastfeeding vs. Infant formula feeding	4 (1741)	Malnutrition Growth Respiratory tract infections Diarrhoea	Becquet *et al*. 2007 Mbori-Ngacha *et al*. 2001 Kindra *et al*. 2012 Mbori-Ngacha *et al*. 2001 Venkatesh *et al*. 2011 Mbori-Ngacha *et al*. 2001 Becquet *et al*. 2007 Venkatesh *et al*. 2011 Kindra *et al*. 2012
Breastfeeding with extended ARV prophylaxis vs. breastfeeding with short-course ARV prophylaxis	5 (7956)	Growth faltering Pneumonia Gastroenteritis Meningitis Sepsis	Jamieson *et al*. 2012 Kesho Bora 2011 Kumwenda *et al*. 2008 Coovadia *et al*. 2012 Kesho Bora, 2011 Gray *et al*. 2005 Kumwenda *et al*. 2008 Kesho Bora, 2011 Jamieson *et al*. 2012 Coovadia *et al*. 2012 Kesho Bora, 2011 Gray *et al*. 2005 Kumwenda *et al*. 2008 Jamieson *et al*. 2012 Kumwenda *et al*. 2008 Jamieson *et al*. 2012 Kumwenda *et al*. 2008
Early cessation of breastfeeding vs. standard duration	2 (451)	Growth Prolonged diarrhoea	Arpadi *et al*. 2008 Fawzy *et al*. 2011
Chemically or biologically acidified infant formula vs. standard infant formula	1 (132)	Growth Bronchopneumonia Gastroenteritis	Velaphi *et al*. 2008
Concentrated infant formula vs. standard infant formula	1 (1686)	Growth	Winter *et al*. 2009
Chemical acidified infant formula milk with or without prebiotics and nucleotides	1 (84)	Growth	Cooper *et al*. 2010

#### Risk of bias in included studies

**Figure 2:**
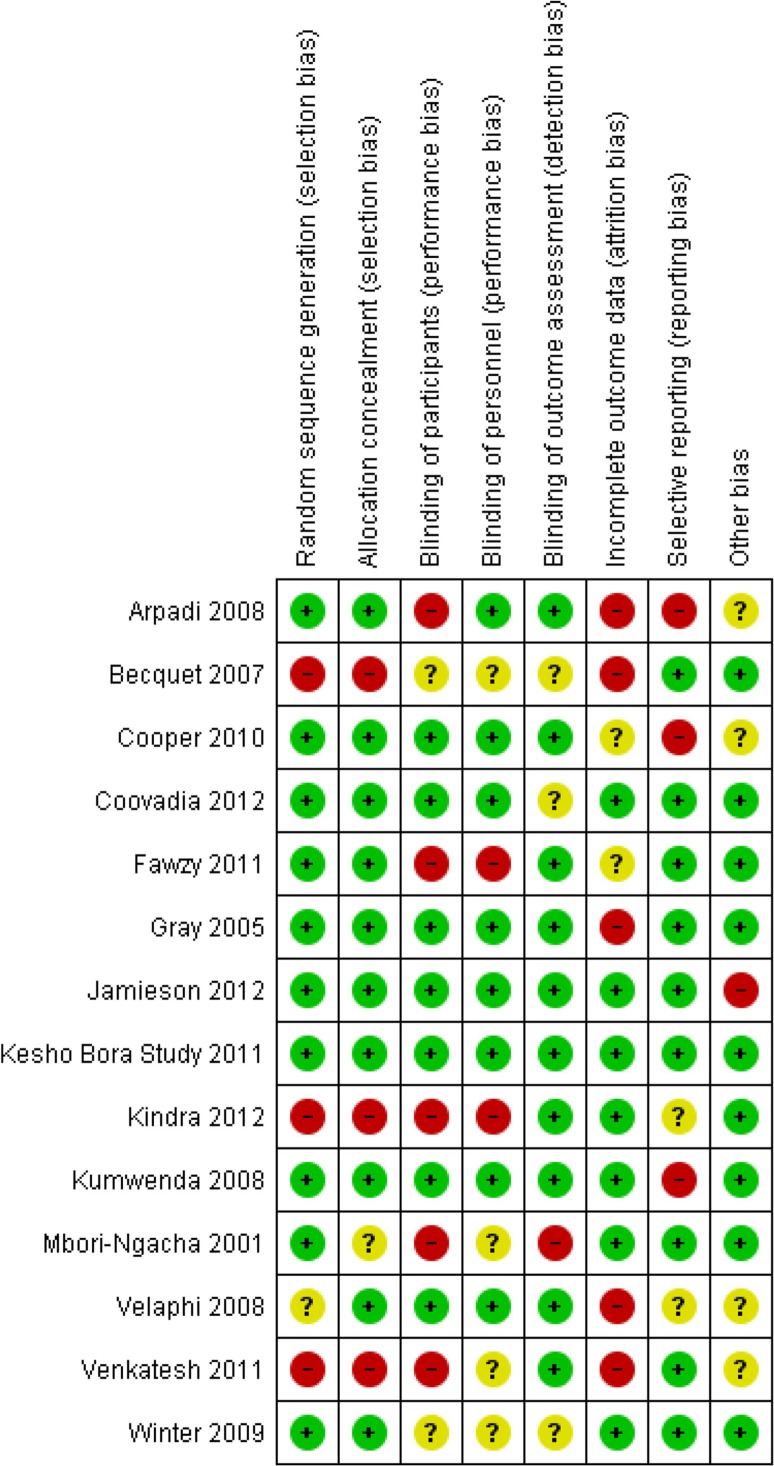
Risk of bias for each domain per study.

**Figure 3:**
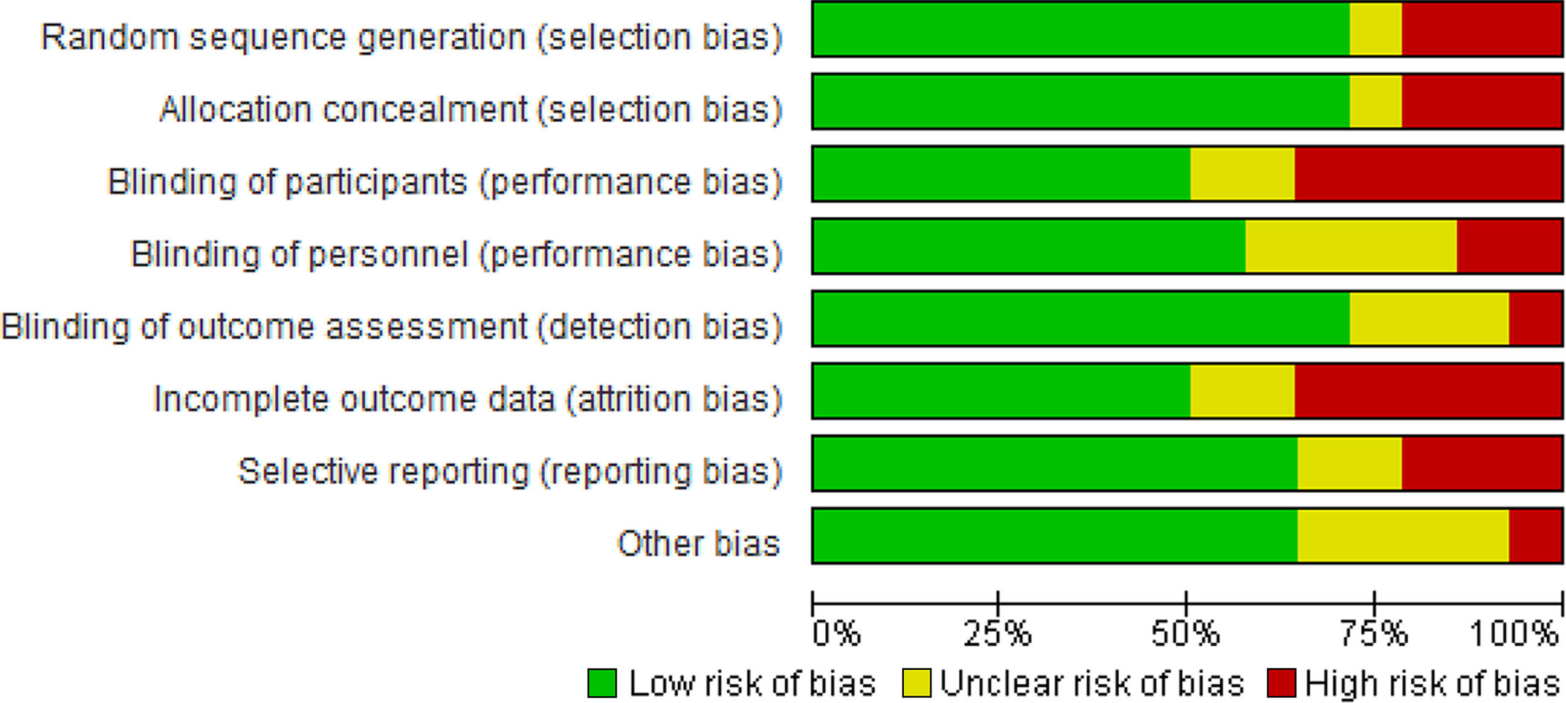
Risk of bias graph for each domain across all studies.

The risk of bias summary presents authors’ judgments on risk of bias in each domain for each study separately (Figure 2), while the risk of bias graph presents the risk of bias in each domain as a percentage across all included studies (Figure 3). A summary of our findings on study methodological quality for each domain follows below.

#### Allocation (selection bias)

Random sequence generation was adequate in 10 studies [9, 11, 23–30]. The method of sequence generation was not reported in one study [31]. Risk of bias was high for three observational studies because participants self-selected into comparison groups [32–34].

Methods of allocation concealment were adequate in 10 studies [9, 11, 23–27, 29–31] . One study did not report how treatment allocation was concealed [28]. The risk of bias in this domain was high for the three observational studies [32–34].

#### Blinding (performance bias and detection bias)

##### Participants and personnel (performance bias)

Seven studies were assessed as having low risk of participant performance bias [9, 11, 24, 26, 27, 30, 31]. Five studies were at high risk because participants were unblinded and it was felt that knowledge of their intervention allocation, rather than the intervention itself, could have affected participants’ outcomes [23, 25, 28, 33, 34]. Two studies were unclear on whether participants were blinded [29, 32]. Risk of personnel performance bias was low in eight studies [9, 11, 23, 24, 26, 27, 30, 31]. Two studies were at high risk because personnel may have treated participants differently through knowing their intervention allocation, thereby influencing the outcomes [25, 33]. It was unclear in four studies whether personnel were blinded [28, 29, 32, 34].

#### Outcome assessment (detection bias)

Risk of detection bias was judged to be low in 10 studies [9, 11, 23–27, 31, 33, 34]. One study was at high risk because outcomes were ascertained through participants’ verbal reports and outcome definitions were relatively subjective [28]. It was unclear whether outcome assessors were blinded in three studies [29, 30, 32].

#### Incomplete outcome data (attrition bias)

Seven reports with an attrition rate below 20% were judged to have low risk of attrition bias [9, 11, 27–30, 33] . Five reports were judged high risk [23, 26, 31, 32, 34], and two studies were unclear [24, 25].

#### Selective reporting (reporting bias)

Protocols were available for five studies [9, 11, 23, 27, 30]. Nine reports were at low risk of reporting bias [11, 25–30, 32, 34] . Three reports were at high risk, because either not all study results were reported at pre-specified time points or the reported outcome was not pre-specified in the protocol [9, 23, 24]. Risk of bias due to selective reporting was unclear in two studies [31, 33].

#### Other sources of bias

Nine studies were judged low risk of other bias [9, 25–30, 32, 33] . One study was at high risk; the Data Safety Monitoring Board recommended enrolment of controls be stopped early because of an apparent intervention benefit [11]. Risk of other bias was unclear in four studies; either baseline characteristics were not compared between study arms, there was a potential for misclassification of exposure status or the role of the funder was not described [23, 24, 31, 34].

### Effects of interventions

Except where specified, results are from combined data from HIV-infected and HIV-uninfected infants. Wherever publications presented findings separately for HIV-uninfected infants we report these. For characteristics of included studies, see Supplementary file.

### 1. Breastfeeding versus formula feeding

One RCT [28] and three prospective cohort studies [32–34] compared growth and non-HIV infections outcomes between breast- and formula-fed HIV-exposed infants. We report the RCT and cohort studies separately.

Mbori-Ngacha *et al*. randomly assigned mother-infant pairs to breast or formula-feeding groups [28]. Cumulative HIV-infection rates by two years of age were 37% and 21%, respectively.

Becquet *et al*. compared infants whose mother chose breastfeeding with rapid transition to formula feeding after four months of age to infants whose mothers chose exclusively formula feeding [32]. HIV transmission rates at 18 months were 5% and 1% among breast- and formula-fed infants, respectively.

Kindra *et al*. compared outcomes of infants whose mothers elected to either breastfeed or formula feed. By six weeks of age, HIV transmission rates were 7.9% and 4% among breast- and formula-fed infants, respectively [33].

Venkatesh *et al*. compared rates of infant hospitalizations associated with infectious morbidity among infants whose mothers elected to breastfeed or formula feed [34]. They documented HIV transmission rates of 18.4% and 13.2% among breast- and formula-fed infants, respectively by three months.

### Outcomes

#### Growth

Kindra *et al*. found no difference in *z*-scores between breast- and formula-fed infants at nine months of age [33].

#### Malnutrition

Mbori-Ngacha *et al*. defined malnutrition as a weight-for-height *z*-score value 2 SD below the mean. Becquet *et al*. defined malnutrition as an observation of either no change or a decrease in anthropometric measurements between study visits. Neither study found a statistically significant difference in malnutrition risk between breast- and formula-fed infants (RR=0.63; 95% CI=0.36 to 1.12) and (HR=1.35; 95% CI=0.93 to 2.0) [28, 32].

#### Respiratory tract infections

Mbori-Ngacha *et al*. do not describe how upper respiratory tract infections were defined [28]. The trial found no difference in rates of respiratory infections between breast- and formula-fed infants (HR=1.00; 95% CI=0.90 to 1.11) [28].

Becquet *et al*. defined acute respiratory infection as cough, fever, and focal pulmonary findings [32]. Venkatesh *et al*. used WHO International Classification of Disease (ICD-10) criteria to classify respiratory infections associated with hospitalizations [34]. The pooled estimate from these observational studies suggests a lower incidence of respiratory infections in breast than formula fed infants (HR=0.65; 95% CI=0.41 to 1.00) [32, 34]. After adjusting for HIV status, breast-fed infants were 40% less likely to develop respiratory infections (HR=0.60; 95% CI=0.36 to 0.98) [32].

#### Diarrhoea

Diarrhoea was defined as the passage of three or more watery stools per 24-hour period for at least two days. Mbori-Ngacha *et al*. found no difference over two years between breast- and formula-fed infants either when including both HIV-infected and HIV-uninfected infants (HR=1.11; 95% CI=0.91 to 1.43) or in HIV-uninfected infants alone (HR=1.11; 95% CI=0.83 to 1.43) [28]. Venkatesh *et al*. reported similar findings (HR=0.50; 95% CI=0.15 to 1.70) as Mbori-Ngacha *et al*. Becquet *et al*. and Kindra *et al*. differ from Mbori-Ngacha *et al*. and Venkatesh *et al*. Both studies found that breast-fed infants were at lower risk for diarrhoea (RR=0.31; 95% CI=0.13 to 0.74) [33], the risk was significantly lower for breast-fed infants after adjusting for HIV status (HR=0.74; 95% CI=0.57 to 0.97) [32].

### 2. Breastfeeding with extended ARV prophylaxis versus short-course ARV prophylaxis

A clinical adverse event is defined as any health-related reaction or effect experienced by a study participant. Serious clinical adverse events (SAEs) in infants were assessed as safety endpoints in studies comparing differing postnatal ARV prophylaxis. Five studies compared the incidence of SAEs between infants exposed to different combinations of extended and short-course ARV prophylaxes during breastfeeding [9, 11, 26, 27, 30]. We use the term “extended ARV prophylaxis” to refer to interventions involving ARVs given for longer duration than the (short-course) peri-partum prophylaxes that were standard of care at the time the studies were conducted. Important assumptions were made for interventions in this comparison. First, Jamieson *et al*. and the Kesho Bora Study included maternal ARV interventions in their studies. Since mothers were breastfeeding while receiving the intervention, infants would be ingesting ARVs in breast milk. On this basis, we felt these interventions could reasonably be compared with ARV interventions administered directly to infants. This assumption is supported by findings of Shapiro *et al*., that concentrations of NVP, lamivudine and ZDV in breast milk of HIV-positive women receiving HAART are similar to or higher than their serum concentrations, and that infant serum NVP concentrations were sufficient to inhibit HIV-1 replication [35]. Second, the studies by Jamieson *et al*. and Kumwenda *et al*. each tested two extended ARV regimens against a standard short-course regimen. We felt that the two extended ARV interventions in each study were sufficiently similar to combine the results for comparison with the short-course ARV group.

Four studies [9, 11, 27, 30] used standard Division of AIDS toxicity tables to grade the severity of SAEs. One study [26] used the WHO ICD-10 criteria. All five studies reported rates of SAEs without stratifying by infants’ HIV status.

Coovadia *et al*. randomly assigned infants who had received six weeks of once-daily NVP to continue a once-daily NVP prophylaxis or placebo until six months of age [30]. Infants were followed-up until 18 months of age. At 12 months of age HIV transmission rates were 3.6% in the placebo group and 2.8% in the NVP group.

Gray *et al*. compared ZDV given to infants for the first six weeks of life to single dose (sd) NVP at delivery [26]. Cumulative HIV transmission rates at 12 weeks were 14.3% in the sd NVP group and 18.1% in the ZDV group.

Jamieson *et al*. compared a control group of mothers given sd NVP during labour or at delivery, and mothers and infants receiving ZDV and lamivudine for one week, to two extended ARV groups: postnatal either the mothers received HAART or infants received daily NVP until 28 weeks of age [11]. At 48 weeks of follow-up, HIV transmission rates were 7% in the control group and 4% in both extended ARV groups. We combined the extended ARV groups to allow pairwise comparison with the control group.

In the Kesho Bora Study [27] mothers received HAART until weaning or a maximum of 6.5 months post-partum (extended ARV group) or ZDV during pregnancy plus sd NVP at onset of labour (short-course ARV group) [27]. By one year of age 5.4% of infants in the extended ARV group became HIV-infected compared to 9.5% in short-course ARV group.

Kumwenda *et al*. compared sd NVP and ZDV given to infants for the first week of life (control group) to 14 weeks of NVP (extended NVP group) or 14 weeks of NVP plus ZDV (extended NVP plus ZDV group) [9]. At nine months of age HIV transmission rates were 10.6%, 5.2% and 6.4% in the control, the extended NVP and the extended NVP plus ZDV groups, respectively. We combined the two extended ARV groups for pairwise comparison with the control group.

### Outcomes

#### Growth faltering

**Figure 4:**
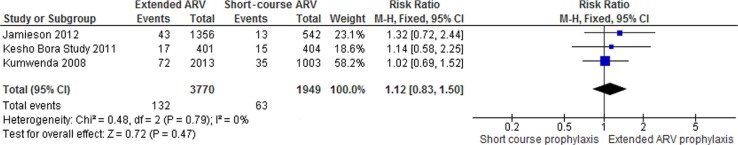
Forest plot of breastfeeding with extended ARV prophylaxis vs. short-course ARV prophylaxis: Growth faltering.

The risk of growth faltering was 12% higher in infants on extended ARV prophylaxis than short-course ARV prophylaxis (RR=1.12; 95% CI=0.83 to 1.50) [9, 11, 27] (Figure 4).

#### Pneumonia

**Figure 5:**
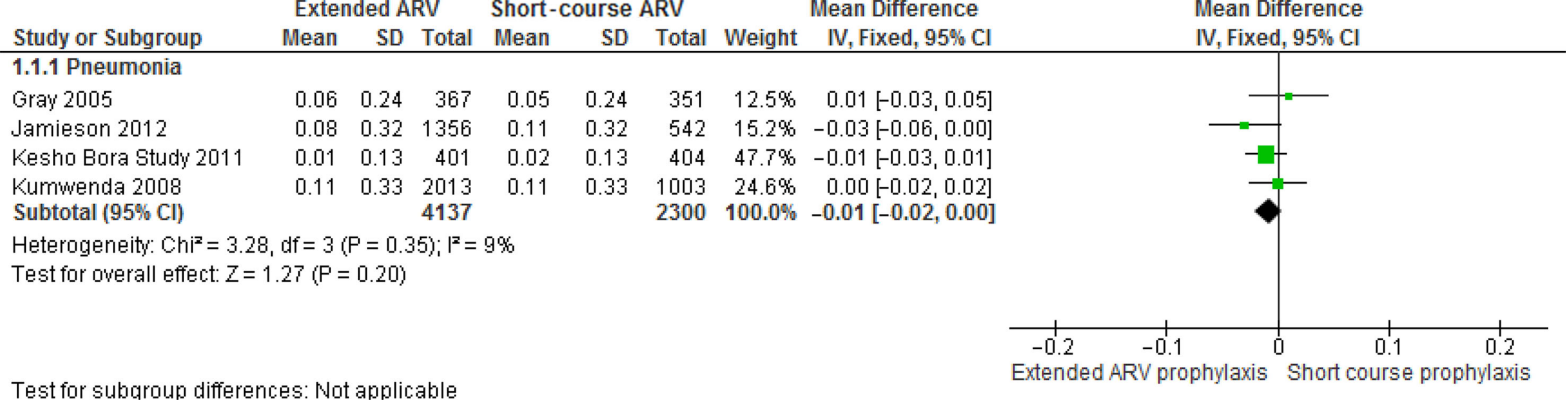
Forest plot of breastfeeding with extended ARV prophylaxis vs. short-course ARV prophylaxis: Pneumonia.

The MD in incidence of pneumonia in the extended ARV prophylaxis group was −0.01 (95% CI=−0.02 to −0.00) [9, 11, 26, 27] (Figure 5). The MD was −0.02 (95% CI=−0.03 to −0.00) when we excluded the study with a high attrition rate. Risk of pneumonia was similar between the groups in the Coovadia study [30].

#### Meningitis

There was no difference in meningitis incidence between extended and short-course ARV prophylaxis groups [9, 11].

#### Gastroenteritis

**Figure 6:**
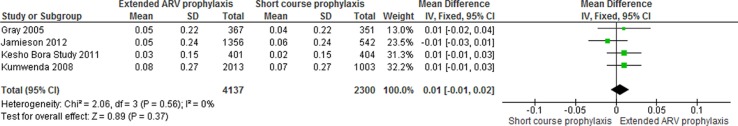
Forest plot of breastfeeding with extended ARV prophylaxis vs. short-course ARV prophylaxis: Gastroenteritis.

There was no difference in rates of gastroenteritis between extended and short-course ARV prophylaxis (MD=0.01; 95% CI=−0.01 to 0.02) [9, 11, 26, 27] (Figure 6). Coovadia *et al*. found no difference in risk of gastroenteritis between the two groups (RR=0.90; 95% CI=0.61 to 1.33) [30].

#### Sepsis

Incidence of sepsis was similar between intervention groups [9].

### 3. Early breastfeeding cessation versus standard duration of breastfeeding

Two reports from a single RCT presented growth and diarrhoeal morbidity outcomes in HIV-exposed uninfected infants whose mothers were randomly assigned to stop breastfeeding at four months (intervention group) or to continue breastfeeding for as long as they wished, with the median duration being 16.2 months (control group) [23, 25]. HIV transmission rates were 21.4% and 25.8% in the intervention and control groups, respectively.

### Outcomes

#### Growth

Weight-for-age *z*-scores at two years of age were similar between infants stopping breastfeeding early compared to continuing for a longer duration (MD=0.12; 95% CI=−0.10 to 0.34) [23].

#### Prolonged diarrhoea

Diarrhoea lasting for at least seven days was defined as prolonged [25]. During the 7–24 months age period, the odds of having an episode of prolonged diarrhoea when breastfeeding was stopped early were almost twice that of breastfeeding for a longer duration (OR=1.70; 95% CI=1.28 to 2.26).

### 4. Chemically or biologically acidified formula versus standard formula

Velaphi *et al*. compared infectious morbidity and growth between HIV-exposed uninfected infants receiving chemically or biologically acidified formula and those receiving standard formula for the first four months [31].

Infants were randomly assigned to four groups:

1. Non-acidified (standard) whey-adapted starter formula
2. Chemically acidified standard formula, where acidification was achieved through addition of L(+) lactic acid
3. Chemically acidified standard formula with *Bifidobacterium lactis* CNCM I-3446 added
4. Biologically acidified standard formula, where acidification was achieved through bacterial fermentation

We combined the two chemically acidified formula groups and the biologically acidified formula group for pairwise comparison with the standard formula group.

### Outcomes

#### Growth

*Z*-scores were calculated based on growth charts from the Centre for Disease Control and Prevention (CDC) and were presented up to four months of age. Head circumference-for-age *z*-scores were significantly higher in infants who received acidified formulas compared to infants who received standard formula (MD=0.31; 95% CI=0.15 to 0.48). The study found no significant differences in WLZ-scores (MD=0.09; 95% CI=−0.16 to 0.34), LAZ-scores (MD=0.08; 95% CI=−0.15 to 0.30) and WAZ-scores (MD=0.18; 95% CI=−0.05 to 0.41) between study groups.

#### Bronchopneumonia and gastroenteritis

The authors do not describe how infectious outcomes were defined. Incidence of bronchopneumonia (MD 0.12; 95% CI=−0.03 to 0.27) and gastroenteritis (MD −0.07; 95% CI=−0.17 to 0.02) between birth and six months of age were similar between infants on acidified formula and those on standard formula.

### 5. Concentrated formula versus standard formula

Winter *et al*. assessed growth in HIV-exposed uninfected infants randomly assigned to receive either 87 kcal/100mL concentrated infant formula or 67 kcal/100mL standard formula [29].

### Outcomes

#### Growth

*Z*-scores were calculated using the 2000 National Centre for Health Statistics paediatric growth references. Mean WAZ-scores were significantly higher for infants on concentrated formula than standard formula (MD=0.12; 95% CI=0.04 to 0.20). We found no significant differences in WLZ-scores (MD=0.11; 95% CI=−0.01 to 0.23), LAZ-scores (MD=0.03; 95% CI=−0.06 to 0.12), and head-circumference-for-age *z*-scores (MD=−0.03; 95% CI=−0.11 to 0.05).

### 6. Chemically acidified formula with or without prebiotics and nucleotides

Growth and infectious morbidity were compared in HIV-exposed, uninfected infants on chemically acidified formula alone and with prebiotics and nucleotides [24]. Infants were randomly assigned to three study groups and followed-up until six months of age:

1. Chemically acidified formula (control)
2. Chemically acidified formula with prebiotics (a blend of short-chain and long chain fructo-oligosaccharides)
3. Chemically acidified formula with prebiotics and nucleotides (a blend of cytidine, uridine, adenosine and guanosine monophosphates)

Chemical acidification was achieved as in [31]. We combined outcomes from infants in Groups 2 and 3 to allow pairwise comparisons with Group 1.

### Outcomes

#### Growth

*Z*-scores were calculated using the 2000 CDC growth charts. The primary study report presented summary data and corresponding 95% CIs in graph format. We estimated mean *z*-scores and SDs from the graphs.

Mean WAZ-scores (MD=0.08; 95% CI=−0.15 to 0.31) and LAZ-scores (MD=−0.14; 95% CI=−0.39 to 0.1) were similar in all groups.

## Discussion

### Summary of main findings

We reviewed findings from seven RCTs and three cohort studies evaluating the effects of various postnatal interventions for PMTCT of HIV.

From our meta-analysis, breastfeeding appears to decrease the risk of respiratory infections by 35%, when infant feeding mode is self-selected and when not considering infant HIV status. However, this finding was not supported by the only RCT reporting on this comparison. There is moderate quality evidence that the risk of respiratory infection remains lower (by 40%) in breast-fed infants through to two years of age, after adjusting for infant HIV status [32].

The evidence from this review is inconsistent on the effect of breast versus formula feeding on diarrhoeal morbidity. In three observational studies, breastfeeding significantly reduced the risk of diarrhoeal morbidity in early life [33, 34], and, of diminished magnitude until the second birthday [32]. We graded this evidence of moderate quality. A randomized trial found no significant difference in diarrhoeal morbidity between breast- and formula-fed infants up to two years of age [28]. This trial was not powered to test equivalence of diarrhoeal morbidity in the two arms. Therefore, it is important to avoid interpreting the lack of statistical significance as evidence of equivalent risk. Breastfeeding is expected to reduce diarrhoea incidence. There are a few possible explanations why this was not observed in this trial. HIV transmission was higher among breast-fed than formula-fed infants, probably obscuring the protective effect of breastfeeding. However, even when results from HIV-uninfected infants were analysed separately, no significant difference was found. A limitation of performing this type of sub-analysis is that the comparison groups are no longer “as randomized,” therefore not necessarily comparable in baseline characteristics, thus possibly obscuring the true effect of breastfeeding. In our opinion, the most likely explanation is that as 30% of mothers assigned to the formula group had breastfed their infants [28], some protective effect of breast milk occurred in the formula group.

In the only RCT comparing breastfeeding versus formula feeding, the risk of malnutrition was 37% lower in breast-fed infants. Though not statistically significant, the reduction in risk may be clinically important because the 95% CI includes strongly protective values at the lower limit and excludes values indicating appreciable harm from breastfeeding at the upper limit. Also, high HIV transmission rates in breast-fed infants would be expected to attenuate any protective effect of breastfeeding, pulling the estimate towards the null. On the other hand, high rates of non-compliance in the formula-feeding group could be confounding this result. This evidence is judged of low quality. Further research comparing the nutritional outcomes of breast- versus formula-fed infants of HIV-positive mothers is warranted.

We found moderate quality evidence that breastfeeding with extended ARV prophylaxis is associated with fewer pneumonia episodes compared to breastfeeding with short-course ARV prophylaxis in HIV-exposed infants. The causal explanation remains unclear. If evidence of this association continues to accumulate, further investigation to explain the underlying biological mechanisms should be prioritized. However, high HIV transmission rates in infants on short-course ARV prophylaxis, especially in studies contributing substantial sample size to our meta-analysis [9, 27], could explain the higher incidence of pneumonia experienced by infants in this group.

Our meta-analysis shows a modestly increased risk for growth faltering among infants in the extended ARV group. This estimate has a wide CI, which includes the null effect. However, there is some evidence that infants exposed to ARV therapy *in utero*, compared to postnatal ZDV, have reduced growth up to two years of age [36]. Therefore, growth of infants exposed to postnatal ARVs should be studied further.

We did not find the rates of any other SAEs to differ significantly between infants in the extended and short-course ARV prophylaxis groups. We do not believe that inadequate follow-up explains the lack of observed differences. In addition, with sample sizes of between 1898 and 5719 for different outcomes, it seems reasonable to conclude that extended ARV prophylaxis does not increase the risk for HIV-exposed infants to experience non-HIV infections outcomes compared to short-course ARV prophylaxis.

## Conclusions

### Implications for practice

Breastfeeding may reduce the risk of diarrhoeal morbidity, respiratory tract infections and malnutrition compared to formula feeding in HIV-exposed infants. Extended ARV prophylaxis and formula feeding can effectively reduce or prevent late postnatal transmission of HIV infection (19). The magnitude of absolute benefit of breastfeeding combined with extended ARV prophylaxis may be sufficient to improve survival of children.

The benefits of breastfeeding with extended ARV prophylaxis must be weighed against the risk of HIV transmission through breast milk when making decisions about feeding HIV-exposed infants. Baseline risks, such as maternal viral load, safety of ARVs and sustained adherence should also influence decisions. Uptake of exclusive breastfeeding is reportedly poor in most African settings [37, 38]. Sub-optimal infant feeding practices are likely to modify the effectiveness of breastfeeding with ARV prophylaxis, especially in normal practice settings. Continuous evaluation of these interventions to determine whether their effectiveness remains clinically important should be a priority as these interventions are introduced into clinical practice.

### Implication for research

Large prospective cohort studies with sufficient length of follow-up are justified to investigate the effectiveness of postnatal interventions for PMTCT of HIV in normal practice settings. The studies should include infectious morbidity and infant growth as primary outcomes. Effects of specific infant formulas on growth require further evaluation.
